# Litter sex composition influences plasma prolactin levels but not the melanin‐concentrating hormone immunoreactive neurons in the medial preoptic area of late lactating Long‐Evans rats

**DOI:** 10.1111/jne.70043

**Published:** 2025-05-14

**Authors:** Ammir Y. Helou, Camila de Carvalho, Larissa A. do Carmo, Jackson C. Bittencourt

**Affiliations:** ^1^ Laboratory of Chemical Neuroanatomy, Department of Anatomy Institute of Biomedical Sciences, University of Sao Paulo Sao Paulo SP Brazil; ^2^ Center for Neuroscience and Behavior Institute of Psychology, University of Sao Paulo Sao Paulo SP Brazil

**Keywords:** lactation, neuroendocrine regulation, neuropeptides, reproductive physiology, sex‐specific maternal adaptation

## Abstract

This study examines the influence of litter sex composition on melanin‐concentrating hormone immunoreactive (MCH‐ir) neurons in the ventromedial medial preoptic area (vmMPOA) and on plasma prolactin levels in lactating rats. MCH is a critical regulator of maternal behavior and displays sexual dimorphism within the MPOA, making it an important target for understanding neuroendocrine adaptations in lactation. Prolactin, a pivotal hormone in lactation and maternal care, was also assessed to elucidate its interaction with litter sex composition. Thirty lactating female rats were divided into five experimental groups based on litter sex composition: all‐male (10 male pups), all‐female (10 female pups), balanced control (five male and five female pups), predominantly male (seven male and three female pups), and predominantly female (three male and seven female pups). On post‐partum day 19 (PPD19), the dams were euthanized for biological analysis. Blood samples were collected for plasma prolactin quantification, and the brains were processed to analyze MCH‐ir neurons in the vmMPOA. Results showed no significant differences in food and water intake or the number of MCH‐ir neurons in the vmMPOA among experimental groups. However, significant variation in prolactin levels was observed, with the all‐male offspring group exhibiting the highest levels (mean prolactin level 23.9 ng/mL, *p* < .001), followed by the all‐female group (20.3 ng/mL, *p* < .01), compared to the control group (14.3 ng/mL). Additionally, the all‐male group showed a reduction in body weight gain. These results suggest that although litter sex composition does not alter the number of MCH‐ir neurons in the vmMPOA, it significantly impacts maternal prolactin levels. This differential prolactin regulation may reflect distinct physiological demands or caregiving behaviors imposed by homogeneous litters, which could, in turn, influence maternal energy balance, lactation efficiency, and adaptive maternal responses. Understanding these sex‐specific influences on maternal neuroendocrine function has important implications for comprehending maternal care dynamics and energy allocation during lactation.

## Graphical abstract

Influence of litter sex composition on maternal prolactin levels and MCH‐immunoreactive (MCH‐ir) neurons in the vmMPOA of lactating rats. The sex ratio of litters affects maternal physiology, specifically increasing prolactin levels and altering weight gain patterns, while food and water intake and MCH‐ir neuron counts remain unchanged. All‐male litters increase maternal prolactin and reduce weight gain, indicating higher metabolic demands, while all‐female litters increase prolactin and improve weight recovery. These results underscore the adaptive regulation of maternal energy use in response to offspring sex composition, informing our understanding of maternal caregiving and energy allocation during lactation. Prolactin structure obtained from AlphaFold2.

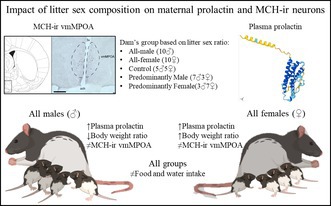

## 1 INTRODUCTION

Melanin‐concentrating hormone (MCH) is a neuropeptide pivotal in integrating endogenous and exogenous information.^1^, ^2^ It is predominantly located in neurons of three diencephalic regions: the lateral hypothalamic area (LHA), the incerto‐hypothalamic area (IHy), and the dorsolateral zona incerta (ZIdl).^3^ This neuropeptide is implicated in a variety of physiological functions, including the regulation of food intake. This phenomenon is evidenced by the increase in MCH mRNA expression during fasting periods in both standard and obese rats and by the subsequent rise in food consumption when MCH is injected into the lateral ventricles, suggesting a direct correlation with body weight regulation.^4^ Additionally, MCH exerts a regulatory function in motivated behaviors, understood as actions intrinsic to the maintenance of homeostasis and the preservation of the individual's or species' life.^1^, ^5^, ^6^, ^7^ Therefore, MCH's influence extends to exogenous elements associated with stress, anxiety, mood, aggression, cognition,^1^, ^5^ and maternal behavior.^8^, ^9^, ^10^, ^11^

Maternal behavior is essential for the survival and continuity of most mammals, being mediated by hormones such as prolactin, which trigger various adaptations in the lactating mother, including the initiation of caregiving instincts for newborns.^12^, ^13^, ^14^, ^15^ These adaptations are closely associated with one of the hypothalamic nuclei, specifically the medial preoptic area (MPOA).^16^, ^17^, ^18^, ^19^, ^20^, ^21^, ^22^ Sexual dimorphism is observed in this area in regards to the expression and production of MCH: while in males, significant expression of MCH in the ventromedial medial preoptic area (vmMPOA) was not observed, in females, there is a progressive increase in the number of MCH‐immunoreactive (MCH‐ir) neurons during the postpartum period, from the fifth to the twentieth‐day postpartum day (PPD5 to PPD20), with a peak on PPD19.^11^ The quantity of MCH‐ir neurons in the vmMPOA directly correlates with the number of offspring.^10^ It is also influenced by prolactin levels, which regulate MCH expression during lactation, with 60% of the cells expressing MCH also expressing mRNA for prolactin receptors and PSTAT5 (transcription factor activated through prolactin receptor activation).^23^, ^24^

In the behavioral context, maternal care in rodents encompasses activities such as nest building, licking, transporting, clustering, and regrouping of offspring.^7^, ^25^, ^26^ Male and female offspring exhibit distinct physiological and behavioral needs, which the mother perceives differently.^27^, ^28^, ^29^ For instance, male offspring require a longer duration of exposure to anogenital licking, a crucial behavior for the initiation of urination and defecation processes, as well as for sexual maturation and the stimulation of testosterone production^30^; this behavior persists from the second day after birth until approximately the eighteenth day.^28^, ^29^ Additionally, male offspring vocalize more than females, and male mice and rats emit more vocalizations in the presence of females or urine‐containing pheromones. These vocalizations are also used to communicate in social contexts. For example, isolated pups may emit high‐frequency sounds (around 40 kHz) to signal their location to the mother and be reconducted to the nest.^31^, ^32^, ^33^, ^34^

Kokay et al.^23^ hypothesized that the transient appearance of MCH‐producing cells during lactation might be regulated by prolactin. They observed that approximately 60% of *Pmch* and *Prlr* mRNA through in situ *hybridization* protocol colocalized and further demonstrated using bromocriptine—a dopaminergic agonist that inhibits prolactin production—that MCH production is directly related to prolactin. Late lactating dams treated with bromocriptine on PPD19 had a reduced number of MCH‐ir cells in the MPOA, while prolactin treatment induced approximately 80% colocalization between MCH and phosphorylated STAT5 (pSTAT5)‐ir cells.^23^

A common approach when investigating neuroplastic mechanisms in the maternal brain involves manipulating litter size, as litter size has been shown to correlate with the number of MCH‐ir neurons in the vmMPOA of lactating rodents.^10^, ^35^ Building on this evidence, we aimed to investigate whether the sex composition of the litter similarly influences MCH‐ir neurons in this brain region, given the distinct energetic and behavioral demands associated with nursing male versus female offspring. Additionally, considering the established regulatory relationship between prolactin and MCH, we examined whether litter sex composition impacts maternal plasma prolactin concentrations during late lactation. We hypothesized that dams nursing all‐male litters would exhibit an increased number of MCH‐ir neurons in the vmMPOA and elevated prolactin levels compared to dams nursing all‐female or mixed‐sex litters.

## 2 METHODS

### 2.1 Experimental groups

For this study, we utilized 40 Long‐Evans lineage animals (*Rattus norvegicus*), comprising 30 females (aged 90 days/P90 at the start of the experiments and weighing approximately 280–340 g) and 10 males as mates. The females were mated using the harem method, wherein three females were allocated with one sexually experienced male per cage, and litter standardization occurred on the first postpartum day (PPD1). The animals were accommodated in polypropylene cages (30 × 40 × 18 cm) within a controlled environment (22 ± 1°C) under a 12‐h light–dark cycle (light from 7:00 a.m. to 7:00 p.m.), with water and rat chow provided ad libitum. The methodology had been evaluated by the Animal Use Ethics Committee (CEUA/ICB) (approved protocol No. 9475080222). The treatment of animals adhered to the protocol of the Brazilian Directive for Care and Use of Animals in Teaching or Scientific Research Activities—DBCA (CONCEA, 2017), prioritizing animal well‐being and the reduction of their suffering.

Five experimental groups were utilized, each consisting of six females (*n* = 6 per experimental group), with each primiparous female housed individually and monitored daily to check for the day of birth (PPD0). On PPD1, the litter was standardized to 10 pups using offspring born on the same day to minimize losses. After the experiment, each group consisted of five (5) animals to appropriately represent the different sex compositions of the litters. The offspring were standardized accordingly:

1. Primiparous all‐male group with 10 male offspring (M).
2. Primiparous all‐female group with 10 female offspring (F).
3. Primiparous control group with five female and five male offspring (C).
4. Primiparous predominantly male group with seven male and three female offspring (PM).
5. Primiparous predominantly female group with seven female and three male offspring (PF).

**FIGURE 1:**
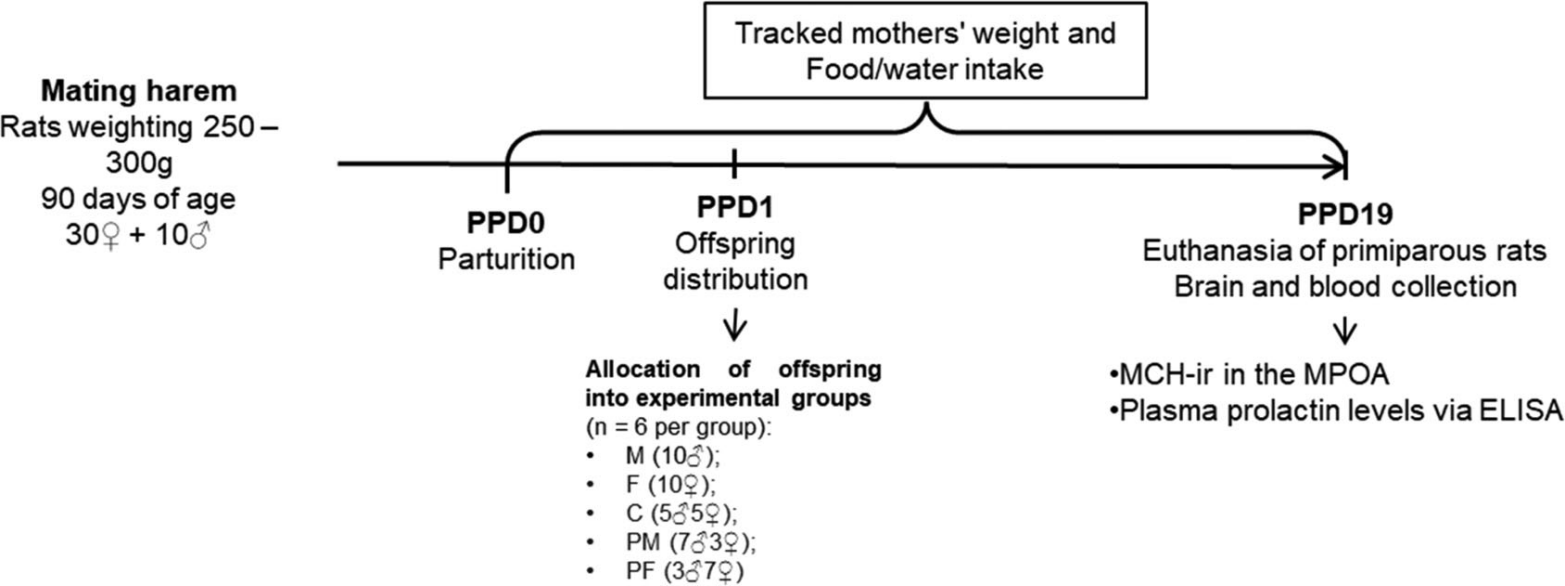
Experimental design and timeline. Monitoring of dams was conducted to establish postpartum day 0 (PPD) on the day of parturition. Litter distribution in the different experimental groups on PPD1. Further information is provided in the text.

Litter standardization was conducted with 10 offspring to increase the number of MCH‐ir neurons in the MPOA at the end of lactation.^10^ The experimental design is depicted in Figure 1.

### 2.2 Dam weight and food/water consumption

The weights of the primiparous rats were recorded on PPD 2, 5, 12, and 19, measured in grams. Concurrently, the weights of the food pellets and water bottles were assessed daily from PPD0 until PPD19. The mothers' weights measured on PPD5, 12, and 19 were also used to calculate the body weight gain ratio to PPD2. To evaluate food and water consumption patterns, the daily monitoring of primiparous rats occurred every morning between 9:00 a.m. and 10:00 a.m. throughout the lactation period from PPD0 to PPD19. The respective weights of chow and water, recorded in grams, were determined by the difference between the measurements of consecutive days.

### 2.3 Euthanasia and biological material collection

On the PPD19 (a.m.), the dams were anesthetized via an intraperitoneal route with an aqueous solution of ketamine (25 mg/mL), xylazine (5 mg/mL), and acepromazine (1 mg/mL) at 0.2 mL/100 g body weight. Once the animal was deeply anesthetized, and performed a transcardiac perfusion with 0.9% saline solution (30 mL/min for 5 min) to clear the vascular bed, and subsequently with 4% formaldehyde with 3.8% borax, pH 9.5 (30 mL/min for 25–30 min), both solutions at 4°C. Before the transcardiac perfusion, blood samples were extracted from the right atrium of each dam. This procedure employed a 3 mL syringe fitted with a heparin‐coated needle. The extracted blood was then deposited into a 500 μL tube and allowed to settle for 30 min. Subsequently, the samples were centrifuged at 5°C at 10,000 revolutions per minute (rpm) for 10 min. Following centrifugation, the separated plasma was preserved at −80°C until being processed.

Brains were collected and post‐fixed for 2 h in the same fixative of formaldehyde 4% with 3.8% borax +20% sucrose solution, then stored in 0.02 M KPBS +20% sucrose until section. A freezing microtome was employed to obtain slices of 40 μm, five series spaced 200 μm apart per section, stored in antifreeze solution (20% glycerol, 30% ethylene glycol in KPBS 0.02 M).

### 2.4 Immunohistochemistry

Immunohistochemistry was performed to label MCH‐ir using the peroxidase method in one of the 5 series obtained. Sections were incubated for approximately 18 h at room temperature in 0.02 M KPBS solution with 0.3% Triton‐X‐100, 2% normal donkey serum (Jackson Laboratories®), and rabbit anti‐rat‐MCH primary antibody (1:30,000—PBL #234), provided by Dr. Joan Vaughan and Dr. Paul E. Sawchenko, Peptide Biology Laboratory, The Salk Institute for Biological Studies, La Jolla, CA, USA. Subsequently, sections were incubated for 1 h in biotinylated anti‐rabbit secondary antibody made in donkey diluted 1:800 to localize the antigen–antibody complex, which will be revealed by avidin‐biotin immunoperoxidase. They were immersed in 0.02 M KPBS solution diluted 1:333 for 1 h. To visualize the antigen–antibody complex, sections were incubated in 3,3′‐diaminobenzidine (DAB), ammonium nickel sulfate, and 0.03% hydrogen peroxide in 0.2 M sodium acetate buffer (pH 6.5) for a maximum of 20 min; the reaction was stopped by transferring the sections to acetate buffer pH 7.5. Subsequently, sections were mounted on gelatin‐coated slides, dried in an oven at 37°C for approximately 18 h, dehydrated in an alcohol series, cleared in xylene, and covered with glass coverslips using DPX.

### 2.5 MCH‐ir neurons quantification count

After labeling, 3–5 coronal slices per animal containing the vmMPOA were selected for quantification, starting at approximately bregma 0.24 mm and ending at −0.36 mm, covering a range of approximately 500 μm. MCH‐ir neurons were quantified using Neurolucida® software integrated with a Nikon Eclipse 80i microscope. Standardization of each brain site was guided by cytoarchitectonic references from Paxinos and Watson.^36^ The total number of neurons across all analyzed sections for each animal was used to calculate the total count, which was then averaged for each experimental group. Data were processed using Excel and Prism software, and the total neuron count from all vmMPOA‐containing sections for each animal was used for inferential statistics.

### 2.6 ELISA for prolactin

For the quantitative analysis of prolactin levels, the enzyme‐linked immunosorbent assay (ELISA) was conducted utilizing the Rat prolactin ELISA kit provided by ThermoFisher Scientific® (catalog #ERA50RB), following the manufacturer instructions (assay range 0.41–100 ng/mL; mean intra‐assay CV = 5.09%).

### 2.7 Data analysis

For statistical analyses, we first checked the normality and outliers of the data using the Kolmogorov–Smirnov test or Shapiro–Wilk test and Grubbs' test, respectively. We then applied either analysis of variance (ANOVA) for parametric data or the Kruskal–Wallis test for non‐parametric data, using the GraphPad Prisma® software (10.2.1) to generate the graphs. All data graphs are presented as mean ± standard error of the mean (SEM), with the statistical significance level set as *p* ≤ .05. Specific statistical analysis is reported in each result section.

## 3 RESULTS

### 3.1 Dams food and water consumption and primiparous rats' weight

**FIGURE 2:**
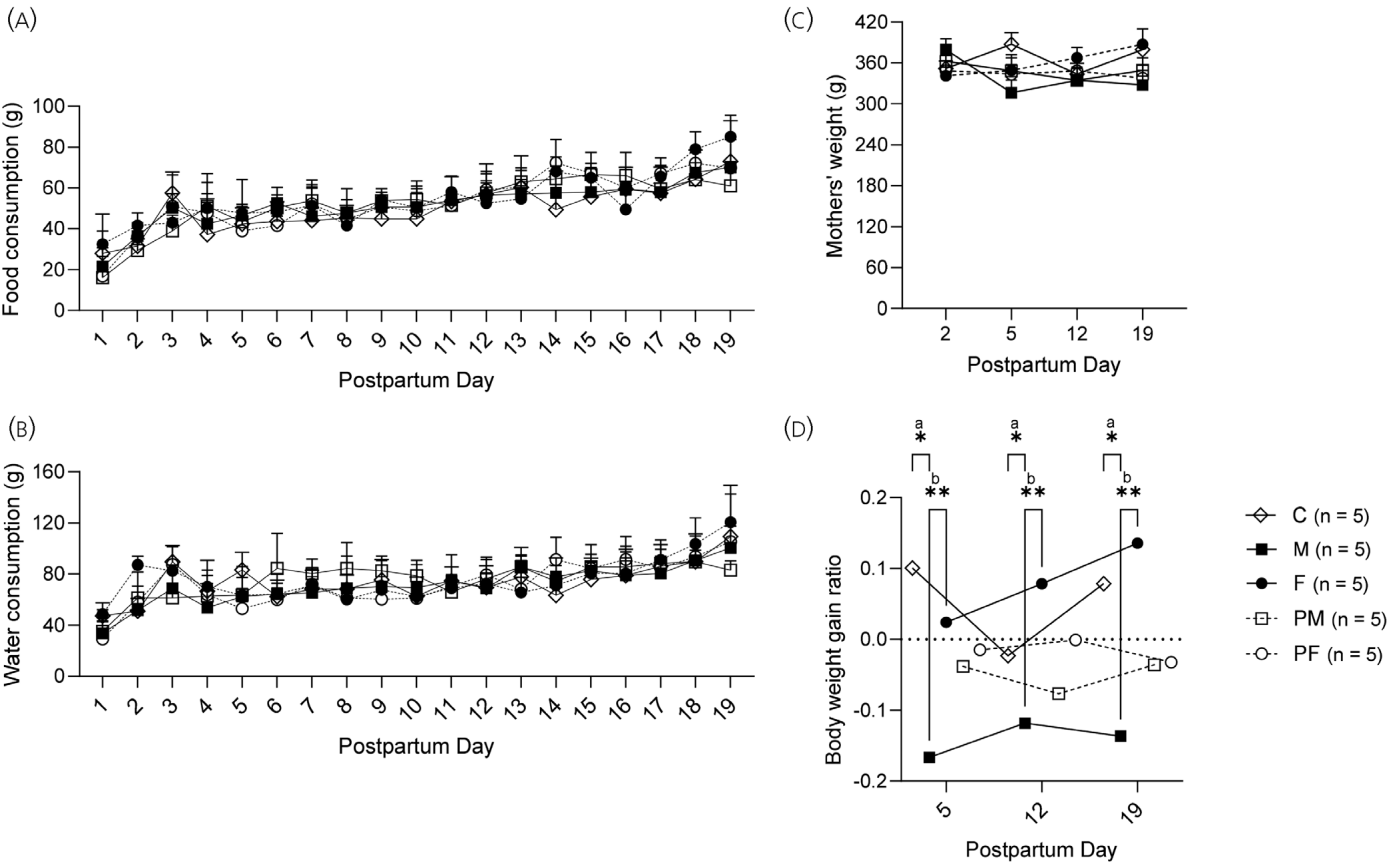
(A) Food consumption (g) and (B) water consumption (g) throughout the lactation period for different experimental groups: Group M (10 male pups), Group F (10 female pups), Group C (5 male and 5 female pups), Group PM (7 male and 3 female pups), and Group PF (7 female and 3 male pups); *n* = 5 per group. (C) Mothers' total body weight (g) at postpartum days (PPD) 2, 5, 12, and 19. Data are presented as mean ± SD. No significant differences were observed between the groups. (D) Specific rate of body mass gain relative to PPD2 for the same groups at PPD5, PPD12, and PPD19 (two‐way ANOVA, post hoc Tukey). *p < .05; **p < .01; “a” for comparisons between M vs. C, and “b” for F vs. C. C, control; M, all male; F, all female; PM, predominantly male; PF, predominantly female.

Throughout the lactation period, food and water consumption were analyzed using repeated measures ANOVA to account for group and time effects. For food consumption (Figure 2A), a significant interaction effect was observed between group and day (*F*(72, 475) = 1.812, *p* < .0002), as well as significant main effects of day (*F*(18, 475) = 45.32, *p* < .0001) and group (*F*(4, 475) = 3.776, *p* = .0049). Post hoc Tukey's multiple comparison tests showed that dams in the F group consumed significantly more food compared to the C group (mean difference = 4.704 g, adjusted *p* < .0019), but this effect was not replicated when analyzing specific day differences. For water consumption (Figure 2B), a significant interaction effect was observed between group and day (*F*(72, 475) = 2.100, *p* < .0001), and there was a significant main effect of day (*F*(18, 475) = 24.91, *p* < .0001). However, there was no significant main effect of group (*F*(4, 475) = 1.790, *p* = .1296), indicating that overall water intake did not differ significantly between experimental groups. These findings suggest that while temporal patterns in food and water consumption varied across the lactation period, the sex composition of the litters had a notable influence on maternal food intake, particularly in dams nursing all‐female litters, but no significant effect on water intake.

The body weights of the dams, measured on postpartum days (PPDs) 2, 5, 12, and 19, showed no significant differences between groups (Figure 2C). Statistical analysis using two‐way ANOVA revealed no significant main effects for group (*F*(4, 100) = 1.684, *p* = .1595) or time (*F*(3, 100) = 0.4984, *p* = .6842), and no significant interaction between group and time (*F*(12, 100) = 1.405, *p* = .1761). These results indicate that the sex composition of the litters did not significantly impact overall maternal body weight across the lactation period.

In contrast, significant differences were observed in the body weight gain ratio across groups (Figure 2D). A two‐way ANOVA indicated a significant main effect of group (*F*(4, 8) = 11.92, *p* = .0019), while the effect of time (day) was not significant (*F*(2, 8) = 0.6209, *p* = .5615). Post hoc Tukey's multiple comparison tests showed that dams nursing all‐male litters (Group M) consistently exhibited lower body weight gain ratios compared to those nursing all‐female litters (Group F; adjusted *p* = .0089) and mixed‐sex litters (Group C; adjusted *p* = .0201) at all measured postpartum days (PPD5, PPD12, and PPD19). The adjusted p‐values for these comparisons ranged from *p* = .0089 to *p* = .0201, with predicted mean differences between −0.2199 and −0.1925 (Table 1). These findings highlight that nursing male litters imposes greater metabolic demands on dams, leading to reduced weight gain during lactation.

**TABLE 1:** Significant Tukey's multiple comparison tests—body weight gain ratio.

Tukey's multiple comparisons test	Predicted (LS) mean diff.	95.00% CI of diff.	Adjusted *p* value
5:M vs. 5:F	−0.2199	−0.3835 to −0.05619	.0089
5:M vs. 5:C	−0.1925	−0.3562 to −0.02883	.0201
12:M vs. 12:F	−0.2199	−0.3835 to −0.05619	.0089
12:M vs. 12:C	−0.1925	−0.3562 to −0.02883	.0201
19:M vs. 19:F	−0.2199	−0.3835 to −0.05619	.0089
19:M vs. 19:C	−0.1925	−0.3562 to −0.02883	0.0201

### 3.2 MCH‐ir in the vmMPOA and plasma levels of prolactin of the dams

**FIGURE 3:**
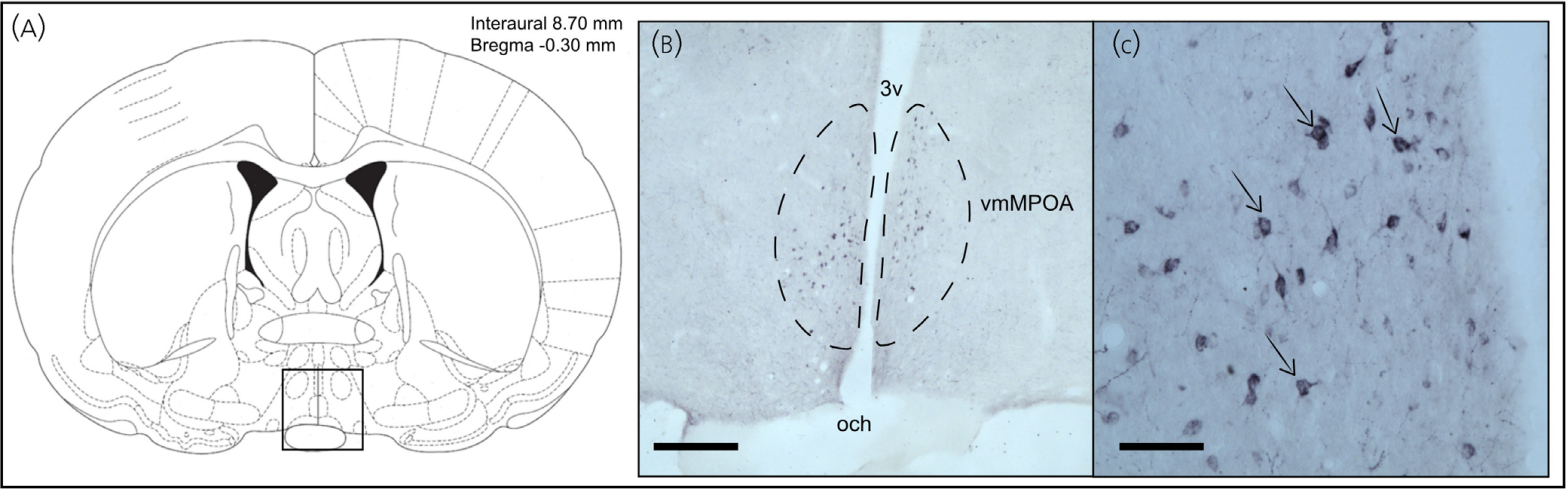
MCH‐immunoreactive neurons in the vmMPOA of lactating dams on post‐partum day 19. (A) Template of a coronal brain section drawing from Paxinos atlas showing the respective area (black rectangle) from Bregma −0.26 to −0.40 mm. (B) Magnified (4×) view of the count area with dashed lines indicating the vmMPOA boundaries. (C) Immunohistochemistry labeling of MCH‐ir neurons (black arrows) in a representative brain slice (20×). Scale bar: B—200 μm; C—50 μm.

**FIGURE 4:**
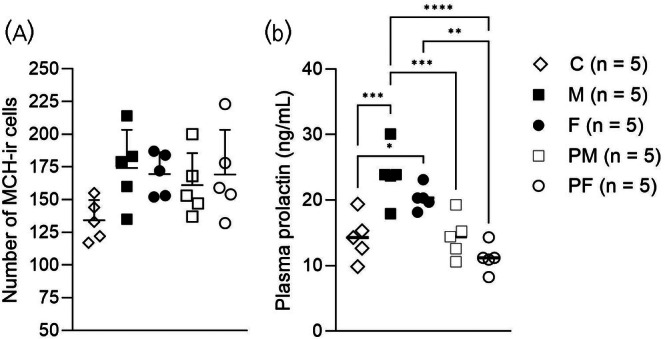
(A) Quantification of MCH‐immunoreactive (MCH‐ir) cells in the MPOA and (B) plasma prolactin levels across different experimental groups. Groups: M (10 male pups per litter), F (10 female pups per litter), C (control with mixed‐sex pups, 5 males and 5 females per litter), PM (predominantly male pups, 7 males and 3 females per litter), PF (predominantly female pups, 7 females and 3 males per litter); *n* = 5 per group. MCH‐ir cells were quantified from approximately 3 slices per animal, including the periventricular area (ordinary one‐way ANOVA). Prolactin levels were measured using ELISA (ordinary one‐way ANOVA, post hoc Tukey's multiple comparison test). Data are presented as mean ± standard deviation (SD). Significant differences between groups are indicated as follows: *p < .05; **p < .01; ***p < .001; ****p < .0001.

The quantification of MCH‐ir neurons in the vmMPOA revealed no significant difference among the experimental groups (Figures 3 and 4A). Statistical analysis using one‐way ANOVA indicated no group effect (*F*(4, 20) = 2.048, *p* = .1262), suggesting that the sex composition of the litters does not significantly influence the number of MCH‐producing neurons in this region.

In contrast, plasma prolactin levels measured on post‐partum day 19 showed significant variation between groups (Figure 4B). Prolactin levels were assessed via ELISA (mean intra‐assay CV = 5.09%) and analyzed using ordinary one‐way ANOVA, with a post hoc Tukey's multiple comparison test, revealing a significant group effect (*F*(4, 20) = 13.48, *p* < .0001). The M group had the highest mean prolactin level (23.90 ± 4.30 ng/mL), followed by the F group (20.31 ± 1.80 ng/mL). Both groups exhibited significantly higher prolactin levels compared to the C (14.30 ± 3.51 ng/mL), the PM group (14.43 ± 3.25 ng/mL), and the PF group (11.17 ± 2.15 ng/mL). Statistical analysis indicated that prolactin levels in the M group were significantly higher than those in the C group (*p* < .001) and the PM group (*p* < .001). Additionally, the F group had higher levels than the C group (*p* < .05) and the PF group (*p* < .01). Tukey's multiple comparisons further detailed significant differences at different group levels: prolactin in the M group differed significantly from both the PM and PF groups, with adjusted p‐values ranging from *p* = .0009 to *p* < .0001 (see Table 2 for complete details). These results suggest that homogeneous male or female litters significantly elevate prolactin levels compared to mixed‐sex litters or control conditions, indicating distinct physiological demands.

**TABLE 2:** Significant Tukey's multiple comparison tests—plasma prolactin.

Tukey's multiple comparisons test	Mean diff.	95.00% CI of diff.	Adjusted *p* value
C (*n* = 5) vs. M (*n* = 5)	−9.599	−15.54 to −3.656	.0009
C (*n* = 5) vs. F (*n* = 5)	−6.011	−11.95 to −0.06856	.0466
M (*n* = 5) vs. PM (*n* = 5)	9.471	3.528 to 15.41	.0010
M (*n* = 5) vs. PF (*n* = 5)	12.73	6.788 to 18.67	<.0001
F (*n* = 5) vs. PM (*n* = 5)	5.883	−0.05947 to 11.83	.0532
F (*n* = 5) vs. PF (*n* = 5)	9.144	3.201 to 15.09	.0014

Together, these results demonstrate that while the sex composition of litters does not impact food and water consumption or the number of MCH‐ir neurons in the vmMPOA, it significantly affects maternal weight gain patterns and circulating prolactin levels. These findings suggest that the metabolic and physiological demands of lactation are influenced by the sex‐specific requirements of the offspring.

In addition to the quantification MCH‐ir neurons in the vmMPOA, we analyzed MCH‐ir neuron counts in the lateral hypothalamic area (LHA), a region primarily associated with feeding behavior and energy balance. Our analysis revealed no significant differences across experimental groups. As these results did not show any group‐specific effects, the data are omitted from this manuscript.

## 4 DISCUSSION

Our study investigated how litter sex composition influences maternal physiology during lactation, with a specific focus on maternal body weight, food and water consumption, circulating prolactin levels, and MCH‐ir neuron counts in the vmMPOA. While significant differences were observed in prolactin levels and maternal weight gain patterns, food and water consumption and MCH‐ir neuron counts did not vary across groups, underscoring the nuanced metabolic and neuroendocrine adaptations associated with lactation. Given the acute orexigenic effects associated with MCH,^37^ we inferred that variations in the number of MCH‐ir neurons in the vmMPOA would occur among different experimental groups, based on the hypothesis that meeting the nutritional needs of male offspring requires increased caloric intake. Although no significant differences in food and water consumption were found between the groups, the body weight gain ratio calculations revealed significant patterns. Group M consistently exhibited a decrease in body mass gain across all measured PPD, indicating potential stress or resource allocation issues affecting weight gain. By PPD19, group M had the lowest average weight gain compared to all other groups. In contrast, Group F showed a steady increase in body mass gain at each time point, with the highest average gain by PPD19, suggesting more favorable conditions for maternal weight gain. The C group experienced fluctuating weight gain patterns, initially increasing at PPD5, declining at PPD12, and then increasing again by PPD19. This variability suggests that a mixed‐sex litter might lead to fluctuating weight gain patterns due to balanced demands of caring for both male and female offspring, likely due to the higher growth rates and energetic needs of male pups.^35^, ^38^ Group PM consistently showed a decrease in body mass gain, similar to M group, implying that having a majority of male offspring might negatively influence the mother's weight. Group PF had slight decreases in body mass gain. Still, these were not as pronounced as those in the Group PM, suggesting that a female‐biased litter composition might mitigate the weight loss in mothers with male offspring. However, the absence of food intake differences indicates that these demands are met by mobilizing stored energy reserves rather than increased caloric intake, as observed in previous studies of maternal energy allocation during lactation.^39^, ^40^

The findings in our study align with significant changes in white adipose tissue metabolism during late lactation in rodents, as discussed by Anhê & Bordin.^39^ During lactation, maternal rodents experience substantial fat mobilization, with up to a 65% reduction in body fat mass by the 21st day of lactation.^41^ This reduction is accompanied by a corresponding decrease in adipocyte volume, particularly in parametrial and subcutaneous fat depots, which remains low until the 15th to 20th day of lactation.^39^ These changes are facilitated by increased lipolysis driven by adrenaline and sympathetic activity, leading to elevated non‐esterified fatty acid availability for the mammary gland. Additionally, lipoprotein lipase (LPL) activity is downregulated in adipose tissues, further contributing to fat mobilization during lactation. These metabolic adaptations in white adipocyte tissue during lactation, emphasizing the increased lipolysis and reduced LPL activity that support the energy demands of milk production, align with our study's context on MCH and prolactin during lactation, as both hormones are crucial in regulating energy balance and lactation physiology. The increased energy expenditure and changes in adipose tissue metabolism discussed in the manuscript are relevant to understanding the physiological mechanisms underlying lactation and how MCH and prolactin interact to regulate these processes.

While our findings focus on the significant reduction in maternal fat mass during lactation, it is crucial to place this process within the broader metabolic adaptations of pregnancy and lactation. During pregnancy, maternal fat reserves increase substantially due to hormonal changes that promote adipogenesis and energy storage in preparation for the high energetic demands of lactation.^38^, ^40^ This accumulation of fat reserves enables the maternal body to support milk production through enhanced lipolysis and mobilization of stored lipids during lactation. Interestingly, animals on an ad libitum diet often end lactation with higher fat levels than they had prior to pregnancy, despite the extensive fat mobilization occurring during this period.^35^, ^42^ These observations underscore the tightly regulated balance between energy storage during pregnancy and energy expenditure during lactation, reflecting the maternal body's ability to adapt to the metabolic demands of reproduction while maintaining physiological stability. Including this perspective highlights the dynamic and multifaceted nature of maternal metabolic adaptations across reproductive stages.

The sex composition of litters significantly influences maternal physiological recovery postpartum. Previous research indicates that energy demands and caregiving behaviors differ for male and female offspring.^28^, ^29^ During lactation, dams' body weight gradually decreased, and by PPD16, the weight was similar to the unmated control group.^43^ Food consumption did not differ between groups throughout lactation, suggesting that metabolic demands and stress, rather than food intake, significantly influence weight changes. Maternal stress and metabolic demands during lactation can impact substantially on body weight.^12^, ^15^ Higher prolactin levels in mothers with all‐male litters, associated with greater metabolic demands of caring for male pups, align with these observations.^44^ The group exhibiting the highest serum prolactin levels was the all‐male. However, the group with the second‐highest serum PRL levels was the all‐female group, although not significantly different from the all‐male group. With these results, it can be inferred that the sex of the litter influences maternal lactation behavior; thus, the greater the number of males or females, the higher the prolactin levels; according to Hashimoto et al.,^44^ exposure to ultrasonic vocalizations increases prolactin levels and enhances maternal behaviors such as searching, retrieving, and nest building; and it is known that male offspring vocalize more.^44^ Interestingly, mothers with all‐female litters had the second‐highest prolactin levels, despite lower expected metabolic demands compared to all‐male litters. This observation may reflect other behavioral or hormonal adaptations, such as the need to maintain balanced caregiving or to compensate for sex‐specific differences in offspring behavior.

The sex composition of litters appears to play a pivotal role in shaping maternal physiological adaptations, particularly in the context of prolactin levels and maternal energy allocation. It has been previously demonstrated that litter composition influences maternal care behaviors, including licking and grooming, which are critical for offspring development.^45^ Additionally, sex‐specific demands from male and female offspring have been shown to modulate maternal metabolic responses, potentially driving variations in weight loss or gain during lactation.^38^ Notably, mothers nursing all‐male litters often face heightened metabolic demands, as evidenced by increased energy expenditure to support the rapid growth trajectories of male pups, while female‐dominated litters may place comparatively lower metabolic strain on the dam.^35^ These findings highlight the complexity of maternal physiological adaptation and the need for comprehensive studies examining milk composition, energy balance, and maternal care behaviors across different litter compositions. Integrating such data could further elucidate how maternal prolactin levels are finely tuned to meet the diverse demands imposed by offspring sex composition.

The findings of Kokay et al.^23^ offer a foundational perspective on the role of prolactin in regulating MCH expression during lactation. They showed that MCH‐positive cells express prolactin receptors and respond significantly to prolactin, highlighting a direct influence of circulating prolactin levels on MCH production. Their use of bromocriptine, a dopamine agonist that inhibits prolactin secretion, led to a marked reduction in MCH expression even under continuous suckling, demonstrating that prolactin is necessary to maintain MCH expression independently of other environmental factors.^23^ While our study did not find significant differences in MCH‐producing neurons across groups, the elevated prolactin in single‐sex litters (particularly in the all‐male group) suggests that maternal physiological adaptation may involve heightened prolactin release to meet energetic demands. Future research exploring circulating MCH levels and neuronal activity markers, such as c‐Fos, would provide deeper insights into these dynamics.

Our findings regarding elevated prolactin levels in the M group, followed by the F group compared to the C groups, align with the hypothesis that litter sex composition influences maternal prolactin levels. De Winne et al. (2023) provided insights into prolactin's regulation of metabolic processes like thermogenesis in brown adipose tissue (BAT), demonstrating how high prolactin reduces BAT's thermogenic capacity by decreasing uncoupling protein 1 (UCP1) expression.^46^ This shift from heat generation to prioritizing energy conservation for lactation supports our findings of increased prolactin in all‐male litters, potentially reflecting a metabolic adaptation to conserve energy for caregiving and milk production. The differential body weight gains we observed in mothers with all‐male versus all‐female litters further emphasize the varying metabolic demands influenced by prolactin and litter composition, consistent with the insights of De Winne et al. (2023).

Chan & Swaminathan^43^ described prolactin's gradual increase toward mid‐lactation, peaking around day 10, and then declining toward the end of lactation.^43^ Our results show increased prolactin levels in mothers of all‐male and all‐female litters compared to mixed‐sex litters, reinforcing the notion that prolactin plays a crucial role in mediating maternal adaptations to litter composition. Barbee et al. (2022) showed that prolactin receptor activation enhances maternal behavior and stress resilience, suggesting that consistent prolactin signaling might help maintain caregiving behaviors even under increased demand.^47^ This may explain why MCH‐ir neuron counts remained stable across groups despite fluctuating prolactin levels, indicating possible compensatory mechanisms in prolactin signaling that stabilize maternal responses.

Our data also corroborate findings by Gustafson et al.,^48^ who reported that elevated prolactin levels correlate with reduced maternal stress and enhanced caregiving. In our study, higher prolactin levels in single‐sex litters likely represent an adaptive response to higher caregiving demands, consistent with the stress‐buffering role of prolactin described by Gustafson et al.^48^ Furthermore, De Sousa et al.^49^ demonstrated that nutritional challenges during lactation significantly impact prolactin levels and maternal care. The stability in MCH‐ir neuron counts despite variations in prolactin levels in our study could be explained by such neuroadaptive mechanisms triggered by differing nutritional or caregiving demands based on litter sex composition. For instance, Shashikadze et al.^50^ reported significant neuroplastic changes in maternal brains associated with larger litters, emphasizing the neural adaptations supporting increased caregiving efforts. The elevated prolactin levels we found in mothers with single‐sex litters and associated differences in weight gain patterns align with these findings, suggesting neuroplasticity in response to caregiving demands that contribute to maternal physiological adaptation.

The consistent counts of MCH‐ir neurons across groups indicate that the neuroendocrine changes linked to motherhood, particularly in the MPOA, do not vary significantly between maternal groups during the late postpartum period. The MPOA is crucial for regulating maternal behaviors and is influenced by hormonal changes.^19^, ^51^ Our results showed no significant differences in the number of MCH‐producing neurons among groups, suggesting that the fundamental neuroanatomic structures involved in maternal behaviors and energy balance remain stable during this period, irrespective of the litter's sex composition or the mothers' prolactin levels and weight changes. Prolactin levels displayed variation among groups, contrasting with the uniformity in food and water intake and MCH‐ir neuron counts in the MPOA. This discrepancy suggests that other factors, possibly including psychosocial stresses or intrinsic maternal characteristics, may influence prolactin secretion independently of metabolic intake or MCH activity within the MPOA. The stability in MCH‐ir neurons suggests that while MCH plays a role in energy balance and feeding behavior, its role in the MPOA during the postpartum period might be more related to the regulation of maternal behaviors than to the direct modulation of energy intake.

Regarding maternal prolactin levels, variability among groups could be attributed to individual differences in responses to single‐sex litters. A clear pattern emerged where single‐sex litters led to higher maternal prolactin levels compared to mixed or control groups, suggesting that offspring sex significantly impacts maternal hormonal responses. This raises questions about the biological mechanisms underlying these differences and their impact on maternal behavior and offspring development. Further research is needed to elucidate how offspring sex composition influences prolactin levels and to investigate the long‐term effects on both mothers and offspring.

## 5 CONCLUSION

This study demonstrates that litter sex composition significantly influences plasma prolactin levels in primiparous rats on post‐partum day 19, with mothers of all‐male and all‐female litters showing higher prolactin levels compared to those with mixed‐sex litters and controls. This suggests that offspring sex plays a critical role in modulating maternal hormonal responses, likely reflecting specific physiological demands or caregiving behaviors associated with homogeneous litters. The hormonal or pheromonal signals from male and female pups likely affect the mother's neuroendocrine system, emphasizing the need to consider litter composition in maternal physiology studies. Hormonal or pheromonal signals from the pups likely impact the mother's neuroendocrine system, modulating the hypothalamic–pituitary axis (Klampfl et al., 2016; Koe et al., 2014).^52^, ^53^

These findings emphasize the importance of considering litter sex composition in studies of maternal physiology and behavior, as it significantly influences neuroendocrine regulation. However, limitations such as the absence of pup weight data throughout lactation, lack of plasma MCH measurements, no quantification of maternal behaviors, and the relatively small sample size may have influenced the results. Despite these limitations, our study highlights the adaptability of maternal physiology in maintaining energy balance and caregiving under varying physiological states. Moreover, a single‐point prolactin measurement may not accurately represent the hormone's production. It is recommended that future studies take this into account in their design. Future research should further explore the interplay between hormonal changes, neural activity, and metabolic adaptations using more comprehensive methodologies, such as hormonal assays, advanced imaging, and behavioral analysis, to provide deeper insights into maternal care and lactation dynamics.

## AUTHOR CONTRIBUTIONS

**Ammir Y. Helou:** Conceptualization; methodology; validation; investigation; data curation; formal analysis; supervision; visualization; writing – original draft; writing – review and editing. **Camila de Carvalho:** Validation; investigation; writing – review and editing; visualization. **Larissa A. do Carmo:** Visualization; investigation. **Jackson C. Bittencourt:** Conceptualization; methodology; resources; data curation; writing – review and editing; supervision; project administration; funding acquisition.

## FUNDING INFORMATION

The authors declare that financial support was received for this article's research, authorship, and/or publication. Financial support for this study was provided in the form of a grant from the Fundação de Amparo à Pesquisa do Estado de São Paulo (FAPESP, São Paulo Research Foundation; Grants # 2016/02224‐1 and # 2023/02531‐5) and from CNPq, Grant # 404806/2021‐0. We are thankful for the support provided by the Brazilian Coordenação de Aperfeiçoamento de Pessoal de Nível Superior CAPES, Office for the Advancement of Higher Education. Jackson C. Bittencourt is an Investigator with the Brazilian Conselho Nacional de Desenvolvimento Científico e Tecnológico (CNPq, National Council for Scientific and Technological Development).

## CONFLICT OF INTEREST STATEMENT

The authors declare no conflicts of interest.

## PEER REVIEW

The peer review history for this article is available at https://www.webofscience.com/api/gateway/wos/peer-review/10.1111/jne.70043.

## Data Availability

The data that support the findings of this study are available from the corresponding author upon reasonable request.
